# Incidental visual memory and metamemory for a famous monument

**DOI:** 10.3758/s13414-022-02472-9

**Published:** 2022-03-31

**Authors:** Pedro R. Montoro, Marcos Ruiz

**Affiliations:** Departamento de Psicología Básica 1, Facultad de Psicología, Universidad Nacional de Educación a Distancia (UNED), C. Juan del Rosal 10, 28040 Madrid, Spain

**Keywords:** Visual memory, Attention, Metamemory, Monuments

## Abstract

In the context of urban life, some monuments are ecologically relevant landmarks for some people. However, previous research on the topic of incidental memory of everyday settings has relatively ignored how people remember monuments from their environments. The present work examined visual memory (i.e., recall and recognition) and metamemory for the Puerta de Alcalá (“Alcalá Gate” in English), a famous ornamental monument in the city of Madrid (Spain). Despite the monument’s perceptual simplicity, participants showed poor visual memory of it in a recall task (drawings), as only 16% of them correctly drew the monument; moreover, only 45% of the participants correctly recognized it in a four-alternative forced-choice test. In contrast, participants reported higher levels of confidence for both recall and recognition (51.57 ± 20.5 and 79.54 ± 19.6, respectively on a 100-point scale). Importantly, memory performance did not vary as a function of the number of years lived near the monument or of the self-reported contact frequency (familiarity) with the monument. The current findings have relevant implications in understanding the link between visual attention, memory, and metamemory in real-world settings.

Though often reliable and accurate, human memory is also fallible. For example, sometimes we cannot recover important data which we absolutely need (e.g., the password to access to our smart phone) despite using it recurrently. Paradoxically, there are other times where we are able to accurately remember some irrelevant information regarding something we have seen only once without much interest (e.g., the name of a supporting character in a *forgettable* movie). Although the relationship between the number of exposures to stimuli and an enhanced memory of them seems obvious, several striking previous results have shown that repeated contact with information does not necessarily lead to accurate memory (see Castel et al., 2015, for a review).

The pioneering study by Nickerson and Adams (1979) revealed the severe difficulty in both recalling and recognizing the features of common coins, a clear example of ubiquitous objects in our daily life. More recent research has obtained similar results showing startling poor memory performance with other everyday objects such as calculator and telephone keypads (Rinck, 1999), computer keyboards used by typists (Snyder et al., 2014), details of road signs (Martin & Jones, 1998), frequently used elevator buttons (Vendetti et al., 2013), the position of fire extinguishers at workplaces (Castel et al., 2012), covers of textbooks for university courses (Hargis et al., 2018), national flags (Blake & Castel, 2019), or the logo of a well-known brand (Apple logo: Blake et al., 2015; Iancu & Iancu, 2017).

This surprisingly imprecise memory does not seem to be attributable to capacity limitations because most of the everyday items used in the studies are very simple and schematic (e.g., Apple logo or coins). In addition, previous works have reported evidence of the massive capacity of explicit memory to store details of up to 2,500 objects (Brady et al., 2008) or to recognize scenes briefly displayed among hundreds of others (Nickerson, 1965). Instead, hypotheses other than that of an overload in capacity have been proposed (see Castel et al., 2015, for a review). One approach suggests a lack of motivation or insufficient attentional resources to retain salient, recurrent information that is not immediately useful to the current task, such as the location of fire extinguishers (Castel et al., 2012). The term “inattentional amnesia” (Wolfe, 1999) sums up this scenario as follows: scarce memory for specific items, even those frequently repeated, may be the result of selective attention failures, inattentional blindness (Mack & Rock, 1998), or interferences during encoding (Fernandes & Moscovitch, 2000). Another possible explanation, also based on attentional factors, is the effect of habituation to stimuli frequently repeated or under prolonged presentations. Thus, attention is not actively allocated to certain common objects. A concept connected to habituation is “attentional saturation” (Bekerian & Baddeley, 1980; Blake et al., 2015). Owing to the familiarity and the lack of novelty of everyday objects, people stop scrutinizing the details of these items, even more so considering their constant availability in our environments and the ease of access to them.

All of those abovementioned accounts assume that the deficient incidental memory is a by-product of attentional factors; yet specific memory-based factors have been also considered to sketch a more comprehensive explanation. In this line, the role of overgeneralization of visual representations was already suggested by the first works on the recall of spatial orientation of heads on coins (Martin & Jones, 1998; Rubin & Kontis, 1983). The reconstructive nature of human memory facilitates the intrusion of prototypical information that summarizes the regularities of the visual world. For instance, Rubin and Kontis (1983) observed that most of the participants wrongly reproduced the head of President Abraham Lincoln (engraved on the 1-cent USA coins) facing to the left, which suggests that people make use of an average schema of coins based on generic properties—such as the dominance of right-oriented faces in coins—when they have to portray a specific coin. Another example of the inferential processes that operate during the retrieval of information is provided by Blake et al. (2015) in their study regarding the memory of the Apple logo. Notably, one third of the participants erroneously included a *stem* when they drew the logo, suggesting that when they began to evoke with a gist-based schema of an *apple* and then, inferential operations completed the representation of the logo, thereby integrating features from the concept of a real apple (Blake et al., 2015, p. 864).

Both attention-based and memory-based explanations are related to the fact that exhaustive retention of detailed information is not necessary to functionally interact with most everyday objects. Our cognitive system applies a minimum informational principle that prioritizes the indispensable information needed to accomplish the function of the common object in daily life. We do not need to remember the precise location of our floor’s button in an elevator because we can find it quickly at a glance. Similarly, discriminating among several textbooks is simple enough by merely remembering that the cover of the Memory handbook is reddish whereas the Attention textbook is dark blue. Remarkably, the study of incidental memory of familiar objects may be critical to better understand the elusive link between attention and memory in ecological environments, which could have relevant implications for many applied problems (teaching methods, advertising, safety in the workplace, etc.).

Another relevant result obtained by previous studies on memory for everyday settings is related to metamemory processes (Blake & Castel, 2015; Schwartz & Metcalfe, 2017). In particular, an interesting dissociation between visual memory performance and metamemory judgments of recall and recognition confidence has been reported by several studies (Blake et al., 2015; Blake & Castel, 2019; Iancu & Iancu, 2017). Apparently, increased exposure to naturalistic settings does not enhance visual memory performance, but does increase familiarity and confidence ratings, which can lead to a very high degree of overconfidence (Blake & Castel, 2019). These divergences between metamemory judgments and memory performance could indicate that people base their metacognitive evaluations on familiarity with the common stimulus or on the accessibility of inappropriate or partial information—not directly related to the visual appearance of the target stimulus—as shown by basic research on metacognitive evaluation (Glenberg & Epstein, 1987; Koriat, 1993, 1995). Interestingly, the study of metacognition in the context of memory of everyday stimuli could provide researchers with relevant information about our knowledge and awareness of our own memory in naturalistic environments.

In line with Bartlett (1932) and Neisser (1978), Castel et al. (2015) suggested that “the use of more ecologically relevant materials . . . can provide a complementary theoretical approach for studying cognition and have more translational impact” (p. 475). Following this remark, a first step on this line has been recently taken by Murphy and Castel (2021), who examined the accuracy of visual memory of a familiar building (the psychology building at University of California Los Angeles [UCLA]) for a sample of undergraduate students from that university. Interestingly, most of participants overestimated the building’s height to width ratio biased by the horizontal–vertical illusion, despite of the actual cubic dimensions of the building. Another related contribution was the work by Rosielle and Scaggs (2008), who tested the (in)ability to detect changes to familiar scenes from a college campus. Performance in the change-detection task was extremely low, in spite of the large scale of the change (e.g., removing a prominent building from a picture).

In line with those innovative lines of research, the aim of the present work is to study the everyday memory for a well-known ornamental monument. For example, could most New Yorkers remember which hand the Statue of Liberty clutches the torch with? We tried to give a response to similar questions about the city of Madrid, by examining recall, recognition and metamemory for the Puerta de Alcalá (“Alcalá Gate” in English; Fig. 1). The Puerta de Alcalá (PA) is undoubtedly the most famous landmark in Madrid and one of the most well-known in all of Spain. It has been the central topic of popular songs, as well as in the front page of tourist guides and in many logos for several sport events and massive entertainment events. Moreover, the PA is located in a crowded crossroad in the core of Madrid, close to the popular Retiro Park, the City Hall and the fountain of Cibeles (where the Real Madrid supporters’ celebrate their football team’s triumphs).

**Fig. 1:**
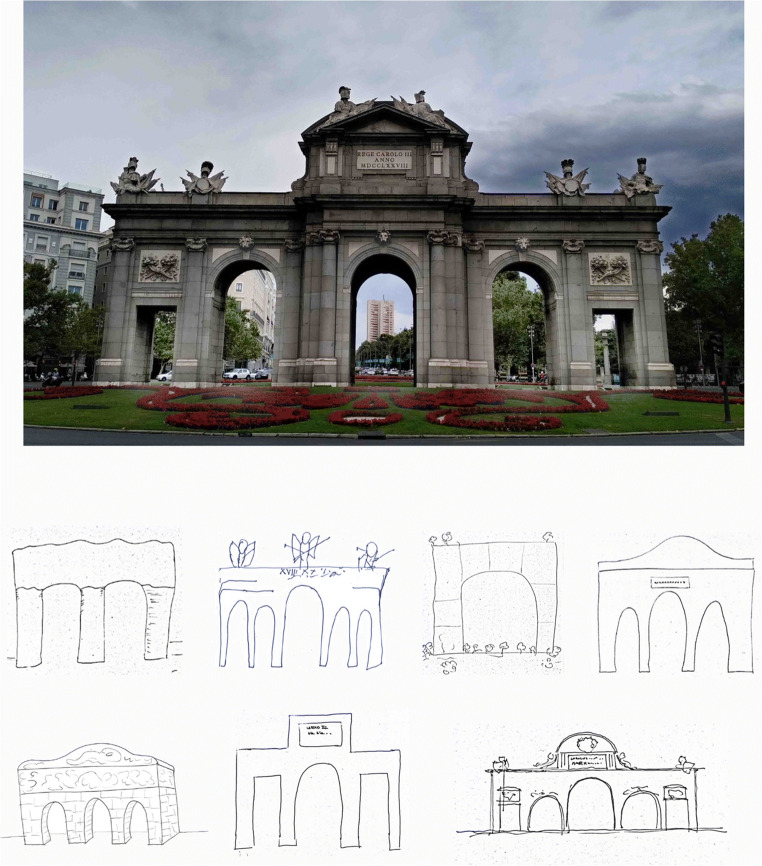
Picture of the Puerta de Alcalá from Madrid (top) and some examples of the drawings from memory by participants in the present study (bottom)

Not surprisingly, some monuments are relevant ecological objects for some people in the context of urban life. They are purposely created and placed in the urban space with the aim of being *seen* by and being delightful for as many people as possible. Emblematical monuments are frequently used as promotional images of cities or countries (e.g., Paris’s Eiffel Tower or the Coliseum of Rome), thus adding an extra virtual contact by seeing them in pictures or videos. Indeed, we can recognize cities by their defining monuments and buildings (the *skyline*; Murphy & Castel, 2021). Additionally, monuments play an important role in guiding navigational behavior in urban contexts (Chan et al., 2012), and even frequently serve as meeting points, especially for crowded areas of the cities. Consequently, the study of monuments as representative everyday objects could provide researchers with a useful tool to further understand both the cognitive and metacognitive processes involved in remembering ecological information in real environments. Hence, for this reason, we considered that making use of a well-known monument from our environment might be especially suitable in order to study the incidental (or passive) memory of everyday objects. At the same time, it might suppose a relevant way to explore the mechanisms of human memory in ecological environments following the line of research opened by Murphy and Castel (2021) and Rosielle and Scaggs (2008). In contrast with previous studies, our everyday setting is not a functional or useful building. The PA is just ornamental or memorial architecture that is preserved to be admired without other use than *seeing* it. In other words, the external design of the PA is clearly the target of every exposure to it. Conversely, the university buildings studied in Murphy and Castel (2021) and Rosielle and Scaggs (2008) were built with clear functional purposes and their external appearance is a secondary visual feature. For this reason, we cannot directly generalize the results obtained by those studies to the visual memory of ornamental monuments and so new data should be collected to explore this topic.

The present study included measures of recall, recognition, metamemory indices and some external predictors of memory (e.g., years living in Madrid) for the PA in order to extend the topic of the incidental memory of familiar, common, everyday stimuli to the class of ornamental monuments, in an attempt to improve the ecological scope of this line of research. The selection of the PA^1^ as target stimulus for our study on passive memory of monuments was highly recommended by two main parameters—namely, (1) the PA is an exceptionally recognized monument that is placed in one of the most crowded points of the city, allowing for a regular exposure for the people from Madrid, by both real and virtual contact; and (2) the PA is a relatively simple, symmetrical, and functionally two-dimensional stimulus that is intuitively easy to remember, as well as effortlessly convertible into a schematic configuration of three Roman arches and two gate—or, even more simply, into a row of five openings in a wall. Subsequently, we recruited a large sample of participants (*N* = 119) with a broad age range (from 19 to 78 years), who had to have lived in the metropolitan area of Madrid for at least the entire past year.

From an intuitive viewpoint, high levels of recall and recognition for a popular and relatively simple ornamental monument, such as the PA, could be predicted, particularly for participants who rate the monument as a familiar object. However, considering the results obtained by previous studies on the memory for common objects, it seems reasonable to hypothesize a relativity poor performance in both recognition and (especially) recall for the PA, while the metamemory judgments will probably exhibit an effect of overconfidence of memory for both tasks.

## Method

### Participants

In total, 119 participants living in the metropolitan area of Madrid (91 females and 28 males; age range: 19–78 years, *M* = 37.1, *SD* = 13.7) participated in the experiment. They were undergraduate students from a second-year course in psychology from the UNED. They received course credits in exchange for their participation. All of the participants had lived in the metropolitan area of Madrid for at least the entire past year. Specifically, the number of years living in the metropolitan area of Madrid ranged from 1 to 66 (*M* = 27.3, *SD* = 17.7) and in Madrid city ranged from 0 to 66 years (*M* = 21.01, *SD* = 18.1). All participants reported normal or corrected-to-normal vision. The experimental procedure was approved by the Local Ethics Committee and conforms to the Declaration of Helsinki.

### Procedure and materials

**Fig. 2:**
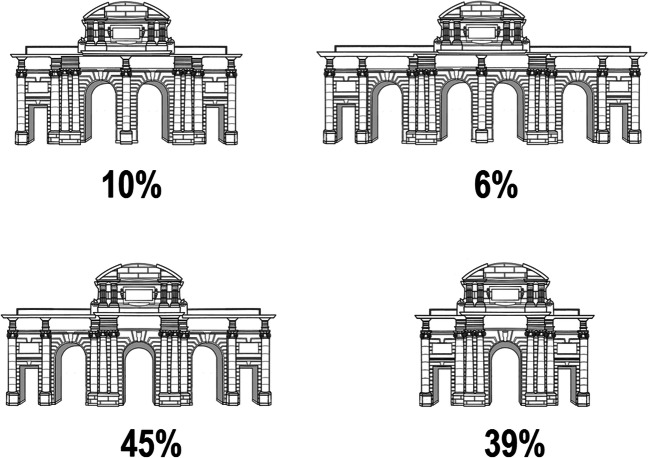
Four possible versions of the Puerta de Alcalá used in the recognition task including the percentage of participants that selected each one. The correct version is displayed on the bottom left panel

The participants were tested individually or in small groups in a quiet room in a single session lasting approximately 30 minutes. They were asked to turn off their mobile phones in order to avoid any search for information regarding the PA. A booklet with five pages and a pencil were provided to them (see Appendix for a detailed description). The participants were then instructed to turn the pages only when the experimenter indicated. The first page included a form requesting relevant personal data—namely, age, gender, place of birth, place of current residence, number of years living in the metropolitan area of Madrid and also in Madrid City. The second page included the subjective rating (from 0 to 100, with 0 indicating extremely low value and 100 indicating extremely high value) of recall confidence, recognition confidence and familiarity, all of them relative to the PA, and displayed in that order. The specific instructions for rating recall confidence, rating confidence recognition and estimating familiarity (based on Snodgrass & Vanderwart, 1980 and Moreno-Martínez & Montoro, 2012) are available in Appendix A. The third page of the booklet was a blank sheet of paper where participants were asked to draw the PA in a 5-minute period. The instructions for this task are also available in Appendix A. The fourth page included eight questions regarding specific features of the PA, although only one was relevant for our objectives (i.e., *How many arches or gateways does the PA have?*), the rest of them were non-relevant filling questions and no analyses of them were conducted (e.g., *How tall is the PA in meters? How wide is the PA in meters?*). The fifth page displayed the following four randomly ordered possible versions of the PA: the real one with five openings (three arches and two rectangular gateways on the sides) and three altered versions with three, four and six openings (all of them with two gateways on the sides and different number of arches). The participants had to rank them in a “scale of reality” from “the most similar or realistic version” to “the less similar or less realistic version” by allotting numbers from 1 to 4, respectively (see Fig. 2). The response was forced, and the numbers could not be repeated.

It is important to highlight that our criterion for testing memory performance was centered on the number of openings of the PA, without discriminating between Roman arches and rectangular gateways. We assumed that the most salient feature of the PA was related to the number of openings shaping its global structure and that the rest of its architectural elements might be considered secondary in discriminating the PA among other similar monuments.

## Results

Figure 2 and Table 1 display the most relevant results obtained in the study. The full data set can be accessed as supplementary materials (https://osf.io/5edas/).

**Table 1:** Percentages of the number of openings or gateways drawn in the recall task

Number of openings drawn	%	Number of participants
One	18.5	22
Two	7.6	9
Three	52.1	62
Four	5.0	6
Five (correct)	16.0	19
Six	0.8	1

### Recall performance

Of the 119 participants tested, only 19 (16% of the sample) accurately drew the PA from memory, where an accurate performance required drawing five openings, irrespective of them being Roman arches or doors with lintels. For example, a drawing with five arches was considered as a hit, and so was a drawing with five doors with lintels. Interestingly, most of the participants only outlined the global structure consisted of arches or openings of the monument and did not include any (or almost any) additional architectural or sculptural elements in their drawings (see Fig. 1 for some examples). Of the 19 participants accurately drawing the PA, only five of them *perfectly* drew the PA (i.e., they drew three central Roman arches and two flanker doors with lintels). The number of openings more frequently drawn was three gateways (62 participants; 52%). Next, 22 participants (18%) drew only one opening; nine participants drew two openings (8%); six participants drew four openings (5%); and only one participant drew six gateways (<1%). The 95% confidence interval of the proportion of hits (.16) ranged between .10 and .24, indicating that the paltry recall was statistically different from zero. A single sample *t* test showed that the overall mean of openings drawn by participants (*M* = 2.95, *SD* = 1.26) was significantly below the correct number (i.e., 5), *t*(11) = −17.7, *p* < .001. Regarding the question about the number of openings or gateways, only 19 participants (16%) answered correctly—the same actual participants who accurately drew the PA. Strikingly, there were another 14 participants (11% of the sample) who answered with a different number of openings from those they had just drawn, although they had failed in both accounts. In fact, the difference between the mean number of openings drawn (2.95 ± 1.26) and the mean number of openings declared (3.19 ± 1.29) was statistically significant, *t*(118) = 3.44, *p* < .05, due to these 14 participants. The consistency of this surprising result should be examined by future research. A detailed inspection of the *inconsistent* participants showed that all of them changed to a higher number of openings than those they had drawn. Six participants changed from one to three openings; two participants from two to three; three participants from three to four; two participants from three to six; and one participant from four to six.

### Recognition performance

The correct PA was chosen as first choice by 45% of the people (i.e., 54 participants out of 119), a rate significantly better than chance (25%), χ^2^(1) = 25.28, *p* < .05. One of the 19 participants who had correctly drawn the PA, chose an incorrect response at the recognition task. Consequently, only 18 participants (15%) were successful at both recalling and recognizing the PA. The version with three openings was selected by 46 participants (39%). The other versions with four and six gateways obtained 10% (12 participants) and 6% (seven participants), respectively.

### Predictors of recall and recognition

The metamemory judgements regarding both recall (*M* = 51.57, *SD* = 20.5) and recognition performance (*M* = 79.54, *SD* = 19.6) reached values much higher than the actual performance in the memory tasks (16% and 45%, respectively), a clear phenomenon of overconfidence. Gamma correlation analyses (Goodman & Kruskal, 1954) showed a significant correlation between recognition confidence and recognition performance (γ = .35, *p* = .02). In contrast, no significant relation between recall confidence and recall level was observed (γ = .15, *p* > .10).

To analyze the number of years living in the metropolitan area of Madrid (YRM) and in Madrid City (YMC) as predictors of recall and recognition, logistic regression analyses were performed including these factors. None of the two factors were reliable predictors of recall nor recognition (all *p*s > .10). However, marginal significance was observed in the cases of YRM as predictor of both recall, χ^2^(1) = 3.15, *p* = .08, and recognition, χ^2^(1) = 3.47, *p* = .06, and YMC as predictor of recall, χ^2^(1) = 3.10, *p* = .08. Owing to the high collinearity between YRM and YMC, *r* = .84, *p* < .05, a new variable reflecting the proportion of YMC with respect to YRM was obtained (i.e., YMC divided by YRM), which was named PYM (range: 0–1; *M* = .73 ± .40), in order to combine both factors in the same model. The correlation between YRM and PYM was only marginally significant (*r* = .17, p < .07), avoiding the problems relative to collinearity. The logistic regression with these two factors as predictors of memory were not significant either for recall or for recognition (*p*s > .10).

The familiarity rated by the participants (*M* = 42.87; *SD* = 24.2) did not correlate with recall (γ = .24, *p* > .10) nor recognition performance (γ = .08, *p* > .10). Similarly, the logistic regression analyses did not detect any significant association between familiarity on its own and memory tasks (*p* > .10). Also, as for models with PYM and familiarity as predictors of recall and recognition no significant regression was found (for both, *p* > .10).

Finally, correlational analyses were performed among the predictors and some significant correlations were observed, namely: YRM and YMC (*r* = .84, *p* < .05); YRM and recall confidence (*r* = .20, *p* < .05); YRM and familiarity (*r* = .40, *p* < .001); YMC and recall confidence (*r* = .23, *p* < .05); YMC and recognition confidence (*r* = .22, *p* < .05); YMC and familiarity (*r* = .43, *p* < .001); familiarity and confidence recall (*r* = .55, *p* < .001); familiarity and confidence recognition (*r* = .36, *p* < .001); confidence recall and confidence recognition (*r* = .57, *p* < .001); and familiarity and PYM (*r* = .25, *p* < .005)

## Discussion

The present work was aimed at extending the study of incidental memory of common objects to ornamental monuments, a class of ecological stimuli in the context of urban life that have been primarily built for being *seen* and admired. Our sample of participants was relatively large (*N* = 119) and included a wide range of both ages (19–78 years) and years lived near to the monument (from only 1 year to up to 66 years), as well as a mixed degree of familiarity with the monument (from 5 to 100). In line with previous related research (Murphy & Castel, 2021; Rosielle & Scaggs, 2008), the results of the present study show that people are extremely inaccurate at both recalling and recognizing a highly popular ornamental monument located in a crowded area of their place of residence, no matter the perceptual simplicity of it and even though the monument is preserved to be *seen* and admired. Remarkably, this poor memory performance did not vary as a function of the number of years people had lived near the monument or of the self-reported familiarity with the monument. In general, the participants overestimated their memory abilities to recover information regarding the monuments. This pattern of results with regard to combining poor memory and overconfidence is very similar to previous studies testing the memory for common everyday objects like coins, logos, or objects from workplaces (Castel et al., 2015) and suggests that incidental human memory—even for everyday stimuli, as simple as they are—is much worse than people believe it to be (Blake et al., 2015).

Which cognitive processes could explain the apparently contradictory pattern of results consisting in poor visual memory of familiar stimuli as well as metacognitive overconfidence? We propose that the contribution of attentional, memory and metamemory factors could account for our main results. As for the attentional domain, an underlying factor may be the lack of necessity of a deep perceptual analysis of monuments to accomplish the typical function of them -at least for most of the people. Apparently, the common function of the PA is *to be looked* at or *to bring about delight in seeing it,* without any specifically behavioral interaction or cognitive demand to be accomplished. As many previous works have underlined, it is not enough merely to “see” an object to accurately remember it, even though the observer looks at the item every day. On the contrary, “noticing” or “scrutinizing” (i.e., the deployment of focused attention, in terms of Rensink, 2000) the visual scene seems necessary to achieve a detailed visual representation of the objects and to encourage a deeper level of processing of the visual stimulation (Craik & Lockhart, 1972; Craik & Tulving, 1975). Attentional mechanisms such as habituation or “attentional saturation” (Bekerian & Baddeley, 1980) induced by familiar objects may also be involved in the results obtained with the PA. The recurrent exposure to a highly available and simple stimulus like the PA make people stop paying attention to the perceptual features of the monument.

**Fig. 3:**
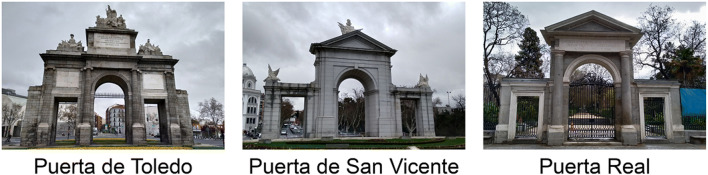
The other three popular gate-shaped monuments placed at popular sites in Madrid

Regarding memory-based factors, an additional account for our results could be the intrusion of overgeneralized information from a prototypical image of “monumental gate” shared by most of the people from Madrid. In the city center of Madrid, there are three other popular gate-shaped monuments placed at popular sites, namely: Puerta del Rey, Puerta de San Vicente, and Puerta de Toledo (Fig. 3). Like the PA, these monuments are sited at crowded points of the city, thereby ensuring a frequent exposure to them for the people in Madrid. Interestingly, these three monuments have a central Roman arch and two doors with lintels on each side and so it is reasonable to consider that people would apply this dominant design to the particular configuration of the PA. This memory process may be related to the classic concept of *assimilation* introduced by Wulf (1922) and Bartlett (1932) to explain the reorganization in memory of visual figures by means of normalization and reduction to a conventional form of the studied material in a serial reproduction task (see Wagoner, 2017, for a review). In support of this hypothesis, our results showed that the number of openings more frequently drawn were the three gateways (52% of participants) and that the three-gateway version of the PA was preferred by 39% of the participants at the recognition test. This was only surpassed by the real PA, which was chosen by 45% of participants. Moreover, an interesting and similar effect of intrusion of prototypical information was presented by Blake et al. (2015) with the gist-based drawings of the Apple logo including a *stem* made by one third of the participants. An open question is which type of overgeneralization process underlay the inaccurate visual representation of the PA in our study. Given this, at least two accounts might be considered if we adapt the main theories of conceptual structure to our topic (see Komatsu, 1992, for a review). One possibility is that participants used an average prototypical schema for “monumental gate” when they recalled the visual features of the PA, in a similar manner to the cognitive mechanisms proposed by the *prototype* theory for semantic processing (Rosch, 1973). In support of that account, Rubin and Kontis (1983) obtained data suggesting that participants had and used a schema for coins that guided their recalls. Another possibility, from an *exemplar-based* approach to semantic categorization (e.g., Medin & Schaffer, 1978), is that people activate representations of exemplars of other previously experienced monumental gates from Madrid rather than a single summary schema of them. Unfortunately, our results did not allow to answer the question and future research should explore this interesting theoretical issue.

Furthermore, regarding memory factors, it should be highlighted that the low performance on recall could be partially fostered by a specific retrieval practice effect. Previous studies on the effect of retrieval practice have found learning to be optimal when the learning assessment employs the same kind of tasks used in previous tests (Duchastel & Nungester, 1982; Endres et al., 2020; McDaniel et al., 1989) in line with a transfer-appropriate processing view (Veltre et al., 2015). Certainly, in daily life, the most usual way of retrieving information regarding the visual representation of monuments could be linked to the direct (or virtual, by means of pictures or videos) contact with the monument. This is similar to a recognition test, whereas recall tasks seem less frequent for monuments, at least for the physical appearance of them. Taking all this into consideration, the question of why our test yielded such a poor recognition performance despite the presumed recognition practice arises. As such, two points should be put forward here. Firstly, in contrast with the cumulative evidence supporting an effect on recall of retrieval practice (e.g., Carrier & Pashler, 1992), previous research has not systematically found similar effects on recognition tasks (Erdelyi & Stein, 1981; Payne & Roediger, 1987; but see Bergstein & Erdelyi, 2008). Secondly, the kind of recognition task we have used seems to mainly focus on the differences among otherwise similar stimuli. Such a procedure would promote analytical processing during the test (i.e., processing focused on the features of the stimulus as the *number* of roman arches; see Hunt & McDaniel, 1993) and such analytical processing would improve the contribution of the *remember* (retrieval) component of recognition as opposed to its *know* (i.e., *familiarity* or *fluency*) component (Mäntylä, 1997). Thus, this brings us back to our previous rationale: the absence of retrieval practice could also account for the poor recognition performance of our participants.

Finally, the paradoxical overconfidence of our participants also deserves some comments. First, in line with previous theoretical proposals and data, our results showed a clear pattern of miscalibration of the metacognitive predictions in the form of overconfidence both at recall and recognition tasks. The frequent contact with the PA probably leads to a disproportionate enhancement of the confidence reported by the participants (Glenberg et al., 1987; Metcalfe, 1993; Metcalfe et al., 1993). In fact, the familiarity rated by the participants was significantly correlated with the number of years living in Madrid and with the confidence at both recall and recognition. A second metamemory factor putatively inducing overconfidence in our study would be the accessibility of overall information relative to both the spatial location in the city map and neighboring urban items. When we search our memory for a solicited monument, it is probable that many clues related to contextual information come to mind. In fact, monuments are relevant landmarks for our cognitive maps of urban environments in support of navigation in the city (see Weisberg & Newcombe, 2018, for a review) or even acting as meeting points at crowded areas of the city. After all, information about location of monuments is significantly more useful for our actions in a city (e.g., navigation or orientation; Chan et al., 2012) than perceptual appearance. Interestingly, laboratory research has shown that the retrieval of contextual nontarget information can spuriously enhance the metamemory judgment (Glenberg & Epstein, 1987; Koriat, 1993, 1995).

In sum, the present work examined the incidental memory of a well-known ornamental monument in an urban context. Our results showing relatively poor visual memory as well as overconfidence in metacognitive evaluations are convergent with previous research on visual memory for common objects from naturalistic environments (see Castel et al., 2015, for a review). This also includes frequently seen buildings too (Murphy & Castel, 2021; Rosielle & Scaggs, 2008). An interesting future line of research could compare current findings obtained in a sample of residents in Madrid with the memory performance of a sample of tourists visiting the city. This comparison might be relevant to contrast predictions from the “attentional saturation” and inattentional amnesia accounts since the tourists should presumably be more motivated to pay attention to the visual appearance of monuments without any distraction or limitation of attentional resources. Undoubtedly, the study of monuments as a representative everyday object provides us with a useful tool for a better understanding of both the cognitive and metacognitive processes involved in the memory of ecological information in real environments, as well as the factors responsible for the dissociation between both measures, in search of the *gates* of human memory.

## Appendix A

Instructions for rating recall confidence:

“*Think about and imagine the Puerta de Alcalá, the monument that is located in the vicinity of the Retiro park in Madrid capital, at the crossroads between the streets of Alcalá, Serrano and Alfonso XII, part of the famous song by Ana Belén and Víctor Manuel. Imagine the Puerta de Alcalá isolated from its context and try to evaluate the degree of confidence in the memory of the characteristics of the Puerta de Alcalá. Think about its shape, color, structure, as well as the percentage of architectural and sculptural elements that you remember from it. To do so, you must use a scale ranging from 0 to 100 according to all the characteristics that you can remember. A value of 0 would mean that you cannot remember absolutely anything about the Puerta de Alcalá, while a value of 100 would mean that you are able to remember with precision and detail absolutely all the characteristics of the Puerta de Alcalá.*”

Instructions for rating confidence recognition:

“*Imagine being shown a set of photographs of monuments, including a photograph of the Puerta de Alcalá. Evaluate, on a scale from 0 to 100, the degree of confidence in your ability to recognize the photograph of the Puerta de Alcalá among a set of photographs of other monuments.*”

Instructions for estimating familiarity:

“*Your task will be to assess your familiarity with the Puerta de Alcalá. By familiarity, we mean the frequency with which you come into contact with the Puerta de Alcalá in your daily life. By “contact” we mean both real contact (visiting and observing the real Puerta de Alcalá) and virtual contact (seeing it on television, in photographs or drawings, or thinking about it or imagining it). To assess familiarity, you have to use a scale between 0 and 100, where the value "100" would indicate almost daily real or virtual contact, while the value "0" would indicate complete absence of contact with the Puerta de Alcalá (that is, to have absolutely never seen, neither in reality nor virtually, the Puerta de Alcalá).*”

Instructions for the recall task:

“*Imagine the Puerta de Alcalá and draw it on the blank sheet of paper below. Imagine the Puerta de Alcalá isolated from its surroundings and just focus on trying to reproduce its structure and architectural elements. You have 5 minutes of time, and when there is 1 minute left, you will be warned so that you can adjust to the time available.*”

Instructions for the recognition task:

“*You will see four different schematic versions of the Puerta de Alcalá which may represent the true Puerta de Alcalá or, instead, may be altered versions or impostures of it. Bellow each figure there is a box to enter the numbers 1 to 4, according to the ‘degree of reality’ of each version. With the number ‘1’ designate the most similar or realistic version to the real Puerta de Alcalá. With the number ‘2’ designate the second most similar or realistic version to the real Puerta de Alcalá. With the number ‘3’ designate the third most similar or realistic version to the real Puerta de Alcalá. And with the number ‘4’ designate the less similar or realistic version to the real Puerta de Alcalá. The answer is forced—you have to order the four versions of Puerta de Alcalá, and the numbers 1–4 cannot be repeated*.”

## Footnotes

1 The selection of the PA as the target monument for our research was initially sparked off by the reading of an offbeat walking guide to Madrid (i.e., “Hidden Madrid”; Besas & Besas, 2007) by the authors of the present work. In the chapter devoted to the PA, Besas and Besas included an illuminating paragraph, which could very well have been written by a researcher on visual memory for everyday settings: “Most Madrileños have passed by the Puerta de Alcalá hundreds if not thousands of times but, strange as it may seem, when you ask them how many gateways . . . the monument has, most will hesitate and be uncertain what to answer. The most common replies are three and four” (Besas & Besas, 2007, p. 280). Strikingly, the phenomenon informally described in this extract is convergent with scientific reports in the context of incidental visual memory of everyday settings.
