# Correction to: Incidental visual memory and metamemory for a famous monument

**DOI:** 10.3758/s13414-022-02505-3

**Published:** 2022-05-10

**Authors:** Pedro R. Montoro, Marcos Ruiz

**Affiliations:** Departamento de Psicología Básica 1, Facultad de Psicología, Universidad Nacional de Educación a Distancia (UNED), C. Juan del Rosal 10, 28040 Madrid, Spain

**Correction to: Atten Percept Psychophys**

10.3758/s13414-022-02472-9

**The author’s proof corrections included answers to the 10 queries listed below; nonetheless, the typesetter did not take any of the answers into account when performing the proof corrections.**

**Q1. Ref. “Koriat, 1993, 1995” is cited in the body but its bibliographic information is missing. Kindly provide its bibliographic information in the list.**

• Koriat, A. (1993). How do we know that we know? The accessibility model of the feeling of knowing. *Psychological Review*, 100(4), 609–639. 10.1037/0033-295X.100.4.609

• Koriat, A. (1995). Dissociating knowing and the feeling of knowing: Further evidence for the accessibility model. *Journal of Experimental Psychology: General*, 124(3), 311–333. 10.1037/0096-3445.124.3.311

**Q2. Ref. “Bartlett (1938)” is cited in the body but its bibliographic information is missing. Kindly provide its bibliographic information in the list.**

• Please, there is a mistake in the year of the work by Bartlett cited in the body. The correct year is “1932”. Please, replace “1938” with “1932” in order to have “Bartlett (1932)”.

**Q3. Ref. “Neisser (1978)” is cited in the body but its bibliographic information is missing. Kindly provide its bibliographic information in the list.**

• Neisser, U. (1978). “Memory: what are the important questions?” in Practical Aspects of Memory, eds M. M. Gruneberg, P.E. Morris, and R.N. Sykes (London: Academic Press), 3–24.

**Q4. Ref. “Goodman & Kruskal, 1954” is cited in the body but its bibliographic information is missing. Kindly provide its bibliographic information in the list**.

• Goodman, L.A., & Kruskal, W.H. (1954). Measures of association for cross classifications. *Journal of the American Statistical Association*, 49, 732–769. 10.2307/2281536

**Q5. Ref. “Rensink, 2000” is cited in the body but its bibliographic information is missing. Kindly provide its bibliographic information in the list**.

• Rensink, R.A. (2000). Seeing, sensing, and scrutinizing. *Vision Research*, 40, 1469–1487. 10.1016/S0042-6989(00)00003-1

**Q6. Ref. “Veltre et al., 2016” is cited in the body but its bibliographic information is missing. Kindly provide its bibliographic information in the list.**

• Please, there is a mistake in the year of the work by Veltre et al. cited in the body. The correct year is “2015”. Please, replace “2016” with “2015” to have “Veltre et al., 2015”.

**Q7. Ref. “Payne & Roediger, 1987” is cited in the body but its bibliographic information is missing. Kindly provide its bibliographic information in the list.**

• The reference “Payne & Roediger, 1987” corresponds to “Payne, D. G., & Roediger III, H. L. (1987). Hypermnesia occurs in recall 794 but not in recognition. *American Journal of Psychology*, 100, 145–795,165. 10.2307/1422400”, which is included in the references list (line 793).

**Q8. Ref. “Bergstein & Erdelyi, 2008” is cited in the body but its bibliographic information is missing. Kindly provide its bibliographic information in the list.**

• Bergstein, J., & Erdelyi, M. (2008). Recognition hypermnesia: How to get it. *Memory*, 16(7), 689–702. 10.1080/09658210802169095

**Q9. Ref. “Koriat, 1993, 1995” is cited in the body but its bibliographic information is missing. Kindly provide its bibliographic information in the list.**

• This query has been already solved in Q1.

**Q10. References [Moreno-Martínez & Montoro, 2012, Neisser, 1982, Payne & Roediger III, 1987, Veltre et al, 2015] were provided in the reference list; however, this was not mentioned or cited in the manuscript. As a rule, all references given in the list of references should be cited in the main body. Please provide its citation in the body text.**

• Please, include “Moreno-Martínez & Montoro, 2012” in the body of the manuscript, in “Procedure and materials” section, line 276 of the manuscript, next to “Snodgrass & Vandewart, 1980” in order to have: “(based on Snodgrass & Vanderwart, 1980, and Moreno-Martínez & Montoro, 2012).

• The query regarding “Veltre et al., 2015” has been solved in Q6

• Please, remove “Neisser, 1982” from the reference list.

• As commented in Q7, the reference “Payne & Roediger III, 1987” corresponds to “Payne & Roediger, 1987” cited in the Discussion section, line 530.

